# Radiomics in Head and Neck Cancer Outcome Predictions

**DOI:** 10.3390/diagnostics12112733

**Published:** 2022-11-08

**Authors:** Maria Gonçalves, Christina Gsaxner, André Ferreira, Jianning Li, Behrus Puladi, Jens Kleesiek, Jan Egger, Victor Alves

**Affiliations:** 1 Center Algoritmi, LASI, University of Minho, 4710-057 Braga, Portugal; 2 Computer Algorithms for Medicine Laboratory, 8010 Graz, Austria; 3 Institute of Computer Graphics and Vision, Graz University of Technology, Inffeldgasse 16, 8010 Graz, Austria; 4 Institute for AI in Medicine (IKIM), University Medicine Essen (AöR), Girardetstraße 2, 45131 Essen, Germany; 5 Department of Oral and Maxillofacial Surgery, University Hospital RWTH Aachen, Pauwelsstraße 30, 52074 Aachen, Germany; 6 Institute of Medical Informatics, University Hospital RWTH Aachen, Pauwelsstraße 30, 52074 Aachen, Germany; 7 Cancer Research Center Cologne Essen (CCCE), University Medicine Essen (AöR), Hufelandstraße 55, 45147 Essen, Germany; 8 German Cancer Consortium (DKTK), Partner Site Essen, Hufelandstraße 55, 45147 Essen, Germany

**Keywords:** precision medicine, head and neck cancer, radiomics, locoregional recurrences, distant metastases, overall survival, CT, multilayer perceptron, XGBoost

## Abstract

Head and neck cancer has great regional anatomical complexity, as it can develop in different structures, exhibiting diverse tumour manifestations and high intratumoural heterogeneity, which is highly related to resistance to treatment, progression, the appearance of metastases, and tumour recurrences. Radiomics has the potential to address these obstacles by extracting quantitative, measurable, and extractable features from the region of interest in medical images. Medical imaging is a common source of information in clinical practice, presenting a potential alternative to biopsy, as it allows the extraction of a large number of features that, although not visible to the naked eye, may be relevant for tumour characterisation. Taking advantage of machine learning techniques, the set of features extracted when associated with biological parameters can be used for diagnosis, prognosis, and predictive accuracy valuable for clinical decision-making. Therefore, the main goal of this contribution was to determine to what extent the features extracted from Computed Tomography (CT) are related to cancer prognosis, namely Locoregional Recurrences (LRs), the development of Distant Metastases (DMs), and Overall Survival (OS). Through the set of tumour characteristics, predictive models were developed using machine learning techniques. The tumour was described by radiomic features, extracted from images, and by the clinical data of the patient. The performance of the models demonstrated that the most successful algorithm was XGBoost, and the inclusion of the patients’ clinical data was an asset for cancer prognosis. Under these conditions, models were created that can reliably predict the LR, DM, and OS status, with the area under the ROC curve (AUC) values equal to 0.74, 0.84, and 0.91, respectively. In summary, the promising results obtained show the potential of radiomics, once the considered cancer prognosis can, in fact, be expressed through CT scans.

## 1. Introduction

More than 650,000 patients are diagnosed with head and neck cancer each year, and 330,000 die from it, making it one of the most-prevalent cancers. This type of cancer has the sixth-highest incidence rate in all of Europe [1,2].

Epidemiological studies show the highest incident rates in central Europe and southwest Asia [3]. This is a complex disease that occurs in different structures: pharynx, oral/nasal cavity, salivary glands, and larynx. The diagnosis and treatment are very challenging, including the variable manifestation of tumours (both primary and recurrent), the complexity of regional anatomy, the minute scale of critical structures, the significant anatomical changes related to tumour response to therapy, and high intratumoural heterogeneity [4].

The standard therapy, depending on the localisation and stage, is surgery, radiation, or radiochemotherapy. In inoperable, recurrent, or metastatic stages of the disease, immunotherapy plays an increasing role. In addition to the clinical examination and biopsy, the preoperative acquisition of 3D medical imaging such as CT scans is indispensable for the correct therapy path assignment [5]. In this context, medical imaging is very important to inform and guide surgeons, radiotherapists, and oncologists, as it allows non-invasive visualisation and assessment of human tissue characteristics and reveals the strong phenotypic differences in cancers before, during, and after treatment [6,7,8].

There are a variety of presentations and clinical outcomes, depending on the location of the cancer. Several strategies are available for treatment, including surgery, radiotherapy, chemotherapy, or a combination of these [9,10]. Even in cancers of the same pathological type (same primary site and same cancer stage), cancer heterogeneity can be observed, the presence of which is strongly associated with a high risk of treatment resistance, progression, metastases, and recurrence [10,11].

Precision medicine is the idea of providing health care, medical treatments, and personalised decision-making tailored to each individual. This concept is not new to medicine as, for example, blood transfusions have been guided by each patient’s blood type for over a century. However, in recent years, precision medicine has been a much-exploited concept and is gaining much popularity, mainly due to the great expansion of biological databases, methods for characterising patients, and improvements in computer tools [12,13]. In the field of oncology, precision medicine is applied as personalised cancer therapy, which means it can determine the best treatment for each patient individually. Its goal is to improve patient outcomes, such as response to treatment and survival without progression of the disease [14,15].

The term “radiomics” refers to the process of quantitatively extracting large and actionable data from medical imaging for further analysis and generation of prognostic values, which is the most beneficial way to complement, facilitate, and accelerate progress towards precision cancer therapy [4,11].

Radiomics applies a number of innovative computational methods to medical image data to convert them into quantitative tissue descriptors. In this context, radiomics, as opposed to biopsies, can help extract information from medical images in a non-invasive way that provides information about the entire tumour. This process allows the characterisation of the phenotype of the tumour. Hundreds of features can be generated/extracted simultaneously from a single lesion. The implementation of radiomics analysis in standard cancer care to support treatment decisions involves the development of predictive models that integrate clinical information, which can be used to assess the risk of specific tumour outcomes [16,17].

This could optimise the therapeutic strategy on a patient-specific basis for head and neck cancer patients. Specifically, therapy could be escalated or de-escalated, and the intensity and frequency of follow-up could be enhanced and potentially lead to improved outcomes in the future [5,18].

## 2. Objectives

The purpose of this research was to verify the effectiveness of translating the field of radiomics into standard care for head and neck cancer, which allows a better characterisation of the cancer phenotype for the construction of predictive models of the head and neck. Radiomics may be a concept for precision medicine, providing improvements in clinical decision-making.

The main objective is to extract features from routine medical images that allow the identification of relevant prognostic factors in the evaluation of the aggressiveness and irregularity of head and neck cancer. Through the construction and development of clinical prediction models that use the learning capacities of Machine Learning (ML) techniques and using both radiomic data and clinical information from patients, the goal was to analyse the three highly relevant prognostic markers in patients with head and neck cancer: risk of locoregional recurrences, evaluate the appearance of distant metastases, and estimate the overall survival rate.

**Figure 1:**
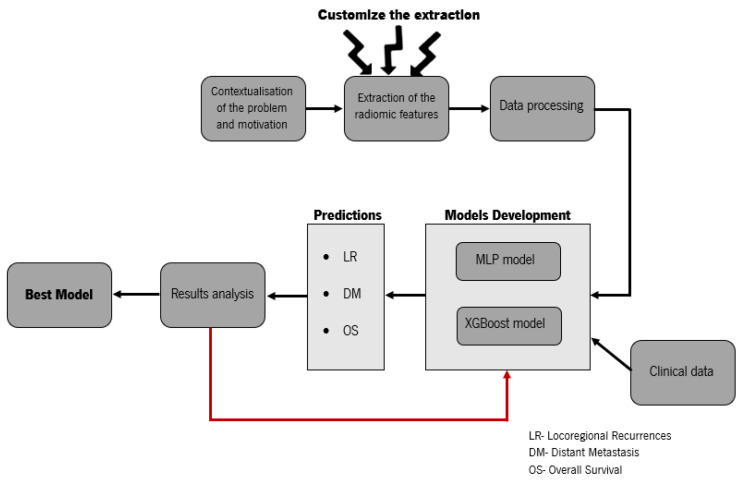
Basic scheme of the workflow for the development of the contribution. The clinical data could include: gender, age, time between diagnosis and start of treatment, and TNM stage. Extraction of radiomic features can be performed with many medical imaging modalities, such as CT.

In Figure 1, we present the basic scheme of the workflow for the development of the contribution of this research. Our study presents new insights into the postprocessing of medical imaging data and the comparison of radiomics with the clinical data. We wanted to investigate whether radiomics could play a key role in predicting locoregional recurrences, distant metastases, and overall survival, in contrast to [19], which used image biomarkers in combination with clinical parameters. This paper also contains parts that are written in a more technical way for easier reproduction.

## 3. Material and Methods

### 3.1. Data and Preprocessing

For this study, a public dataset was used, which is available on The Cancer Image Archive (TCIA) website; the downloading was performed via the NBIA Data Retriever [14]. The information for patients with histologically proven head and neck cancer came from four different institutions in Quebec: Centre Hospitalier de l’Université de Montréal (CHUM), Centre Hospitalier Universitaire de Sher-brooke (CHUS), Hôpital Général Juif (HGJ), and Hôpital Maisonneuve-Rosemont (HMR). All cases were head and neck squamous cell carcinoma (HNSCC).

**Figure 2:**
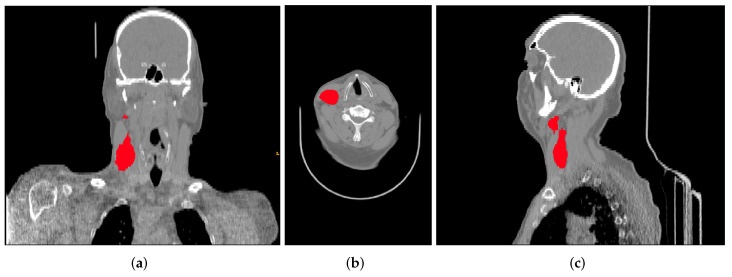
Example of a CT scan from the data collection with the corresponding segmentation of the gross tumour volume, shown in all three planes: (**a**) coronal, (**b**) axial, and (**c**) sagittal.

This data collection contains FDG-PET/CT pre-treatment scans (Figure 2) with a median of 18 days (range: 6–66) before treatment, clinical information (sex, age, primary site, cancer stage, HPV status, among others), and the radiotherapy structure set, plan, and dose, respectively, RTStruct, RTPlan, and RTDose. Part of the tumour segmentation was performed directly on the CT scan of the hybrid PET/CT scanner by oncologists specialized in radiotherapy and used for treatment planning. However, in most cases, the tumour was segmented in a separate CT scan for therapeutic planning and, through the software MIM (MIM software Inc., Clevant, OH, USA), was projected on the FDG-PET/CT scan.

Of the 298 cases available in the dataset, only 183 subjects were included in this study. The reason for excluding cases was that, in 68 cases, we were not able to extract all information and, in the other 47 cases, we had problems matching the intra-case data. The downloaded data are available in the Digital Imaging and Communications in Medicine (DICOM) format, a standard format that enables the development of Picture Archiving and Communication Systems (PACSs) and allows the storage, manipulation, printing, and transmission of medical images. This format supports a wide variety of modules, but for this study, it was decided to use only the information from CT and radiotherapy (RT). For the latter, it is still possible to find a set of “sub”-specific modules: RTImage, RTDose, RTStruct, RTPlan, and RTTreatment [16].

Among other information, RTStruct provides a set of labelled regions of interest (in this dataset, it identifies, for example, the brain, eyes, and neck, among other organs). This module is extremely important for the project as it is where the analysed tumour segmentation is located. Initially, the data were visualised thought the 3D Slicer platform, a software for the analysis and visualisation of medical images, which allows the development of scientific research [20]. The platform was used to understand the structure of the dataset and to analyse both the CT and RT structures.

However, the open-source *PyRadiomics* package, which was used to extract the radiomic features, does not support this file format. Subsequently, the data were converted to Nifti. The conversion was accomplished simultaneously through different tools: the CT and RTStruct images were converted via dicom2nifti [21] and dcmrtstruct2nii [22], respectively. It is important to note that, despite the data conversion, the meta-information of each image (date and time of acquisition, echo time, repetition time, effective echo spacing, coding direction, etc.) was also stored in the Nifti files [23]. The result of applying these two tools was a set of Nifti files: one for each mask/labelled region of interest available in RTStruct and one file for the converted CT scan.

### 3.2. Extraction of Radiomic Features

**Figure 3:**
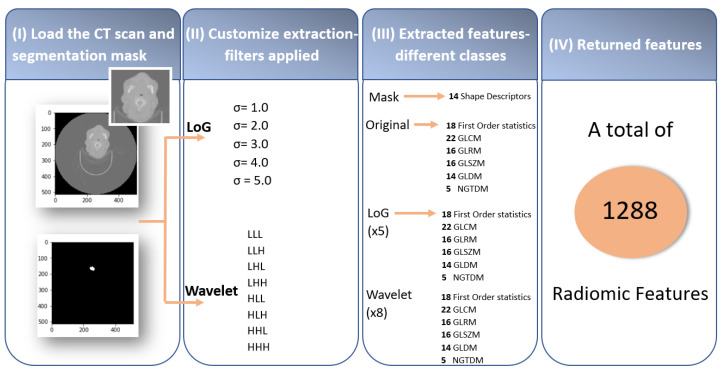
Summary scheme representing the 4 fundamental steps for the extraction of radiomic features. Initially, on the acquired medical images, a segmentation of the ROI is performed, followed by the customisation of the feature extraction from the segmented region by applying filters. Later, the extracted features (e.g., features based on tumour intensity, shape, and texture) are analysed in terms of their ability to predict valuable information for treatment planning.

The extraction of radiomic features was implemented in Python using the open-source package *PyRadiomics* [24]. As represented in Figure 3, this process, when performed through *PyRadiomics*, has four fundamental steps: (I) loading the scans of the medical images and the respective masks with the tumour segmentation (RTStructs), (II) customising the extraction by applying filters, (III) extraction of the radiomic features using the different classes, and (IV) returned features.

The first step was crucial for the rest of the process because, although the dataset contains a segmentation of the gross volume of the lymph nodes and the primary tumour, it was decided to only use the primary tumours’ information. The lymph nodes were not always present, and they were not always metastases.

Afterwards, radiomic feature extraction was performed on the original images, and after, we applied two filters, Laplacian of Gaussian (LoG) and wavelet, to perform the multiscale texture feature analysis of the tumour [25,26].

The LoG filter consists of the junction of the Gaussian filter, whose function is to soften the noise, with the Laplacian operator, which detects the edges and ridges. In this study, five filters with different standard deviations were used (σ = 1.0, 2.0, 3.0, 4.0, and 5.0 mm), corresponding to five filtered images for every input dataset [27,28].

**Figure 4:**
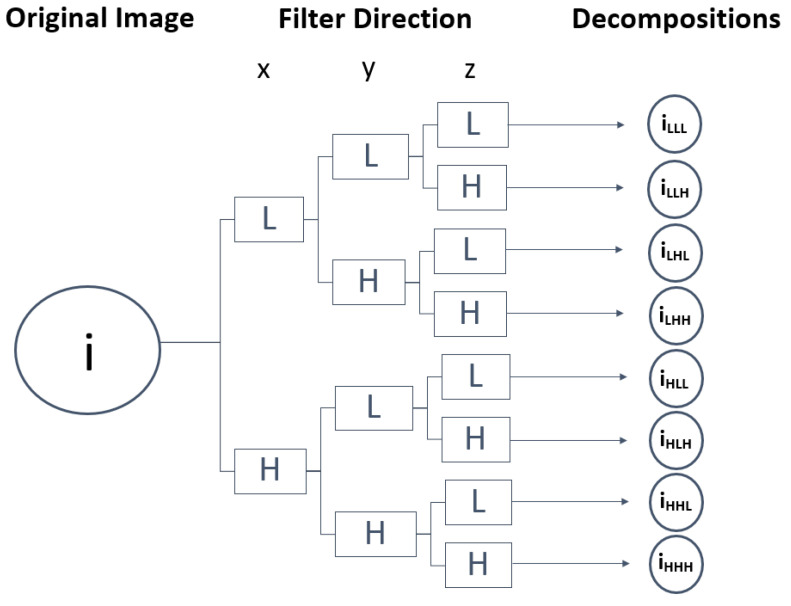
Representation of the wavelet 3D decomposition applied to each CT scan performed in this work. The original image (i) is decomposed into 8 images by applying a directional Low-pass filter (L) and a High-pass (H) filter.

The wavelet filter was used to further study the texture characteristics that best describe the homogeneity of the lesions, leading to the distinction between benign and malignant tumours [26]. The wavelet employs a set of special filters called quadrature mirror filters to recursively decompose the original image into low- and high-frequency sub-bands along the x-, y- and z-axes. In total, from a single image, it is possible to obtain eight new images of the same size as the original. The low-pass filter and high-pass filter being represented by L and H, respectively, from the original image (i), the following wavelet decompositions were obtained: i_LLL_, i_LLH_, i_LHL_, i_LHH_, i_HLL_, i_HLH_, i_HHL_, i_HHH_ [25,26,29]. Figure 4 presents a representation of the wavelet 3D decomposition applied to each CT scan performed in this work, in which the z-axis analysis allows the study of the probabilities of articulation between slices over an image volume composed of multiple slices, i.e., the spatial transitions between voxel values. Through this method, it was expected to increase the sensitivity and specificity of the tumour characterisation, consequently identifying suspicious areas of tumour boundaries that could appear as normal [30,31].

The third step consisted of the extraction of the radiomic features using the different classes. The *PyRadiomics* package comprises a variety of seven classes with different numbers of associated features: 14 shape-based, 18 first-order, 22 Gray-Level Co-occurrence Matrix (GLCM), 16 Gray-Level Run-Length Matrix (GLRLM), 16 Gray-Level Size-Zone Matrix (GLSZM), 14 Gray-Level Dependence Matrix (GLDM), and 5 Neighbouring Gray-Tone Difference Matrix (NGTDM) features. The shape descriptors only characterise the mask; however, the remaining features were taken from the original image: the five LoG filters and the eight wavelet decompositions. In conclusion, a set of 1288 radiomic features was extracted, as represented in the summary scheme of Figure 3. The list of extracted features is presented in Part A in the Supplementary Materials.

### 3.3. Data Processing

Once the radiomic features have been extracted, it is extremely important to process the data in order to build the clinical prediction models. The data are presented in table format, so that, the first concern was to remove the columns that contained unrelated data for the analysis, such as the software versions (PyRadiomics, Numpy, SimpleITK, PyWavelet, and Python), the hash, and the columns containing invariable data. For the radiomic features, a unity-based normalisation by columns was performed, setting all values in the range from 0 to 1. Some changes/adaptations were also made to the data on clinical variables. These data contain both categorical and numerical variables and were, therefore, processed in different manners. Starting with the categorical variables, the first step was to standardise the terms since the information was written differently, for example through uppercase and lowercase or different spacing, and the program understood them as being different variables. The categorical variables already standardised were subsequently converted into dummy/indicator variables. In the implementation of this step, the get_dummies function available in the Pandas library was employed [32]. It is important to note that cases with missing values were treated as another category.

Concerning the numerical variables of the clinical data, the first change was in the content of the columns. The original file contained one column with the time (in days) from diagnosis to the start of treatment and another with the time (in days) from diagnosis to the end of treatment; this second column was changed to the time (in days) of treatment by subtracting the mentioned columns. This modification was applied simply to make the analysis more perceptible. Another particularity of these data is that they contain negative values in columns, whose meaning is supposed to include only positive values, for instance the time (in days) from diagnosis to the start of treatment. The most appropriate method would be to eliminate the patients in which this occurs; however, they are a significant number of cases in relation to the total number of patients. Therefore, it was decided to replace them with the general average over the entire dataset. Finally, the numerical variables were again normalised. Once the data had been properly processed, the last step before the construction and development of the LR, DM, and OS predictive models was to divide the dataset into the test and training cases.

## 4. Head and Neck Cancer Outcome Predictions

### 4.1. Experimental Process and Global Results

This section describes the experimental process to train and evaluate the final models for predicting the risk of locoregional recurrences, assessing the appearance of distant metastases, and determining the overall survival rate of head and neck cancer patients. The public dataset available on the TCIA website used for this study was utilised for similar studies performed by [10,11]. To enable a fair comparative study between the results obtained in this work and in the studies already published, we used the same division into the test and training cases and evaluation metrics. Therefore, the patients from the institutions CHUM and HMR were used for testing and CHUS and HGJ for training. In order to evaluate and understand the results, confusion matrices were constructed, and the evaluation metric applied was the area under the ROC curve (AUC); more detail is given in Section 4.2.2.

In the course of this study, several experiments were carried out to supplement and reinforce the results presented in the current work. Initial studies consisted of developing the predictive models by applying all nine filters available in the *PyRadiomics* open-source package. However, this approach was not very successful, as the models suffered from overfitting and the AUC values were very low. For this reason, it was decided to analyse the interference of each filter, and the conclusion was that the selection of the wavelet and LoG filters led to the best results. In the predictive models of the XGBoost algorithm, this was corroborated by the bar charts containing the 20 features that most positively influenced the construction of the decision trees. Most of these features were the ones that were extracted after applying the selected filters (as will be seen in the following section).

Furthermore, the influence of clinical variables on the predictions was examined. Initially, the models were constructed using only the radiomics features (imaging features) extracted from the CT scans through the open-source package *PyRadiomics*. Afterwards, the clinical data of the patients were added. As explained in Section 3.3, the clinical data contained implausible treatment time information. Therefore, the addition of the clinical variables was performed in two phases: Experiment 1 contained only the clinical data of the patients such as age, gender, and primary site of the tumour, among others, and Experiment 2 additionally contained information about the time, in days, from diagnosis to the start of the treatment and to the last follow-up and the treatment time. As a consequence, three experiments were conducted for each prediction. The list of clinical variables added and their distribution per institution can be found in Part B in the Supplementary Materials (Table S1).

Finally, a comparative study among several machine learning algorithms is presented for each cancer outcome prediction. The models were developed using the following algorithms: multilayer perceptron, extreme gradient boosting, logistic regression, random forest, and decision trees. The AUC values obtained for all predictions in the different algorithms are presented in Table 1, Table 2 and Table 3. These results were from the test data, i.e., the data that were not used in the training phase.

Through the analysis of the tables, it is possible to conclude that the overall advantage of the XGBoost algorithm is evident because it presently dominated the applied machine learning techniques. This algorithm allowed making predictions with the most adequate confidence; its classification was not random, as it was when using some of the other algorithms.

This study reached results supporting the hypothesis that head and neck cancer outcomes can be manifested by extracting radiomics from CT scans. Although the models containing only the imaging features did not present the desirable AUC values, when predicting DMs and OS, they are promising.

Additionally, this work allowed assessing the influence of the patients’ clinical variables on the predictions. Regarding Experiment 1, the addition of clinical data was an asset for LR prediction, as the AUC value increased from 0.5765 to 0.7181. On the other hand, for the prediction of DMs and OS, the addition of clinical data showed no added value, since the predictive power of the models slightly decreased (AUC values decreased from 0.8273 to 0.8246 and from 0.8462 to 0.8369 for DMs and OS, respectively). This means that the clinical data were crucial for improving the LR results, as using only radiomics gave an AUC value close to 0.5. However, in predicting DMs and OS, the clinical data were not advantageous and even slightly detrimental, so using only radiometric features was better for the first experiment.

Experiment 2 obtained the best results for all predictions. The greatest AUC increase in this experiment was observed in OS prediction, and the times, in days, from diagnosis to the start of the treatment and to the last follow-up were the most important clinical features. The presence of distant metastases and the primary tumour site were also very important for this prediction.

The dataset has a problem in the distribution of the labels, as can be seen in Part C in the Supplementary Materials (Figures S1–S3): in all cases, the number of positive instances is always much lower than the number of negative instances. This notwithstanding, our results suggest that there is a chance to predict the presence of locoregional recurrences, distant metastases, and the overall survival rate using radiomic features from the clinical data of the patients.

### 4.2. Deeper Analysis of XGBoost in Experiment 2

#### 4.2.1. Hyperparameters

XGBoost, when implemented through the scikit-learn library, has essentially four classes: XGBClassifier, XGBRanker, XGBFRegressor, and XGBRFClassifier, which are used to solve the classification, ranking, regression, and random forest regression problems, respectively. Given the problem presented in this project, the most appropriate class was XGBClassifier [33]. Concerning XGBoost’s hyperparameters, it is possible to apply a variety of tuning parameters to tree-based learners (learning rate, max_depth, subsample, colsample_bytree, and n_estimators, among others), as well as regularisation parameters to penalise models as they become more complex and reduce them to simple models (gamma, lambda, and alpha) [34]. The “objective” hyperparameter that determines the loss function used was “binary:logistic”, since it is the most suitable for classification problems with probability. After several experiments, the XGBoost hyperparameters and the respective values that gave rise to the highest AUC values in LR, DM, and OS predictions are presented in Part D in the Supplementary Materials (Table S2).

#### 4.2.2. Confusion Matrices and ROC Curves

Various measures can be used to evaluate the predictive performance of a rating model. The most important classification metrics are based on the so-called confusion matrix, which shows for a set of objects how many of them were correctly classified and how many were misclassified. In a confusion matrix, the two columns represent the predicted classes, while the two rows represent the corrected classes. The main diagonal contains the correct predictions, i.e., True Negatives (TNs) and True Positives (TPs), while the incorrect predictions are contained in the secondary diagonal, i.e., False Positives (FPs) and False Negatives (FNs). Most of these measures were developed for binary classification tasks, but they can easily be adapted to multi-class tasks [35].

The AUC is a good estimator of the predictive performance of a classifier, since, for most classifiers, different decision thresholds for determining the class of a new object lead to different performances, and the AUC takes into account the performance of the classifier at different thresholds by calculating the area under the ROC curve, which makes it possible to study the effects of different decision thresholds on the TP and FP rates [35].

**Figure 5:**
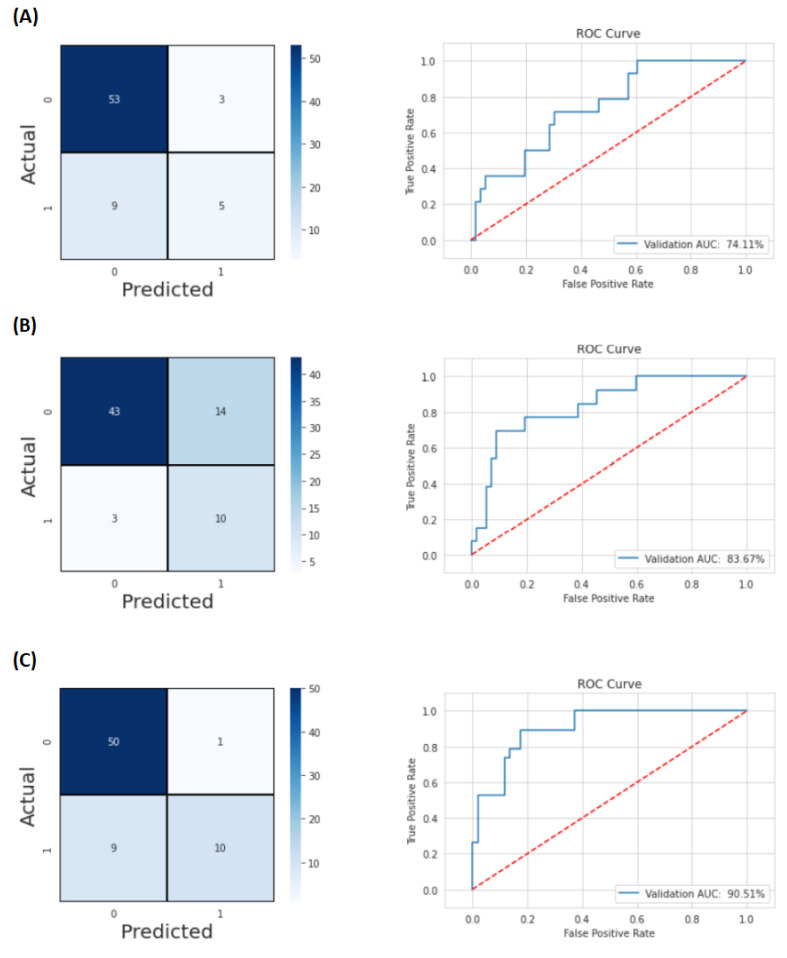
Confusion matrices and ROC curves of the project using imaging features, clinical variables, and the time information—Experiment 2. (**A**) For LR prediction, the AUC is 0.7411; (**B**) the AUC for DM prediction is 0.8367; (**C**) the AUC for OS prediction is 0.9051.

Figure 5 presents the confusion matrices and graphs of the ROC curves obtained in the predictions of LRs, DMs, and OS, respectively, A, B, and C.

#### 4.2.3. Relevant Features

**Figure 6:**
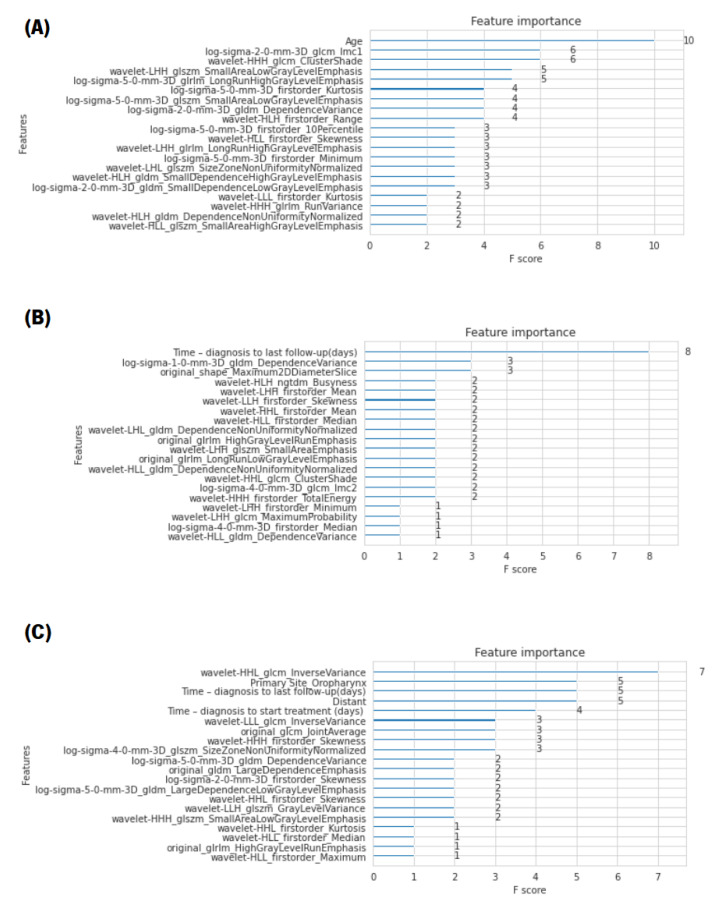
Bar charts with the importance score given to the 20 most-relevant features of the best results. (**A**) For the LR prediction, it is not only noteworthy that the feature with the most influence is age, but also the fact that its F-score is very high, compared to the other features. (**B**) For the construction of the decision trees of the model of DMs, predominantly, features extracted after the application of the wavelet filter were used, demonstrating that the multiscale texture feature analysis of the tumour performed with the application of this filter was decisive in predicting DMs. In addition, the variable with the highest decision weight was the time from diagnosis to the last follow-up. (**C**) Regarding the OS prediction, it can be seen that the presence of distant metastases was a crucial factor for the prediction of OS. Another relevant feature was the primary site of the tumour, more specifically when it was oropharynx. It can also be concluded that the time, in days, from diagnosis to the last follow-up and to the start of treatment were also features with great influence on the construction of the model decision trees.

One advantage of using ensembles of decision trees methods such as gradient boosting and XGBoost is that they can automatically provide estimates of feature importance from a pre-trained model. The importance is defined by a normalised score given to each feature/attribute in order to indicate how useful or valuable that feature was in building the optimal decision trees within the model. The more a particular attribute is used in the decision-making of the tree, the more valuable that attribute will be and, therefore, the greater its importance. The amounts of the different attributes are calculated for each attribute individually; however, they are classified and compared to each other [36]. It is possible to automatically calculate the importance of features in the predictive modelling problem of a trained XGBoost model using the “feature_importances” variable of the trained model. Therefore, an analysis was performed in order to find the most-valuable predictors among the set of features used. For each XGBoost prediction, a bar chart with the score of importance given to each feature, presenting only the 20 most-relevant ones, is presented in Figure 6. The metric used for the analysis of the characteristics was the F-score, which is very appropriate for class imbalance problems. The F-score is the balance between precision, also known as positive predictive value, and recall, also known as sensitivity. Note that precision indicates the percentage of objects classified as positive among all objects that are indeed positive, and recall indicates the percentage of objects correctly classified as positive by the model among all objects classified as positive, also referred to as the TP rate [35].

In this figure, it is not only noteworthy that the features with the most influence for LR and DM predictions were, respectively, age and time (in days) from diagnosis to the last follow-up, but also the fact that their F-scores were very high compared to the other features. Regarding the OS prediction, the presence of distant metastases, the primary site of the tumour, more specifically, when it was oropharynx, and the time (in days) from diagnosis to the last follow-up and to the start of treatment were crucial factors.

Furthermore, from the analysis of the bar chart presented, it is evident that, for the construction of the optimal decision trees, the features that most positively influenced the models were, mainly, the features extracted after the application of the two filters (wavelet and LoG), which performed a texture analysis on multiple tumour scales.

## 5. Discussion

For this study, a public dataset already used for similar studies was used. Hence, in order to evaluate the results obtained in the present work, a comparison of the different projects was made, highlighting the main similarities and differences.

The division into training and test cases was made according to the reference works to allow a more rigorous comparison. However, Vallières et al. [11] included the entire tumour volume, i.e., lymph nodes, as well as the gross volume of the primary tumour, while Diamant et al. [10] only used the central slide of the tumour, i.e., the slide with the greatest number of tumour pixels; in the present study, only the gross volume of the primary tumour was considered. It was decided not to include lymph nodes as the patterns of growth, ingrowth, and necrosis are similar for all types of carcinomas regardless of tumour diversity (as in head and neck cancer). Furthermore, lymph nodes may only be reactively enlarged and not contain any tumour.

Furthermore, the whole process for building the predictive models was different. In Vallières et al.’s study, three radiomic feature sets were considered: features extracted from PET, CT, and a combined set containing both modalities. For each set, an “initial feature” was defined, and all possible logistic regression models of order two were created. Bootstrap resampling was then performed for each of these models to calculate the AUC 0.632 + bootstrap. The remaining feature that maximised this value when combined with the initial feature was selected. Thereafter, to find the best model, the process was repeated cyclically. Finally, the prediction models were combined the imaging scans and clinical variables of the patients through random forest classifiers.

The study by Diamant et al. was specifically benchmarked against Vallières et al.’s study, acting as a complement to that mentioned reference. In this new approach, only CT scans were analysed, and an end-to-end Convolutional Neural Network (CNN) designed de novo was used to predict DMs, LRs, and OS. However, it was only the model for DMs that was found to be significantly better than the benchmark study, so the resulting outcome of the DM CNN model was combined with the benchmark model.

In this work, we proposed a different strategy. The extraction of the radiomic features from the CT scans was carried out on the original images and after the application of the LoG and wavelet filters to perform a multiscale texture feature analysis of the tumour. The final models for each cancer outcome prediction were employed through several machine learning algorithms, with emphasis on MLP and XGBoost.

In addition to the different methodologies, the present study also introduced a major change when clinical variables were added, as it analysed the influence of the time information (time from diagnosis to the start the treatment, treatment time, and time from diagnosis to the last follow-up). The results revealed that this association was an improvement for the success of the study. However, in the reference studies, these variables were excluded.

In order to conduct a more detailed and correct comparison, the analysis of the results of the study by Vallières et al. was divided into two stages: using only the information relative to CT scans and the final results containing PET and CT. The following Table 4 presents the best AUC values obtained in the present study, in the study by Vallières et al. in both stages, and finally, in Diamant et al.’s study of the LR, DM, and OS predictive models.

Our work was based on a public dataset collection, for which we were not able to extract all information for every case. Among the 298 cases available in the dataset, only 183 were included in the construction of the predictive models in this study. The lack of information for certain variables was worsened by this fact, since there were variables studied with zero cases (see the example in Part B regarding the clinical variables). This limited the conclusion of our results, which, however, other researchers would also face with these datasets.

Therefore, it would be expected that the AUC values would be lower compared to the other studies that used the original dataset with all the information. Nevertheless, as shown in the table, the results achieved with our approach were comparable at worst, or better in many cases. The LR and OS predictions showed better results than Vallières et al., not only when compared under the same conditions (using only information from CT scans), but also compared to their overall results with information from the PET and PET/CT scans. In fact, only the prediction of DMs did not present as much success, as its AUC values were slightly smaller. This could be caused by eliminating some patients from the dataset, which might be the most significant/most prominent cases for this prediction.

## 6. Conclusions

Head and neck cancer comprises a large and diverse range of tumours with a complex aetiology and pathogenesis. This cancer presents a great treatment challenge as it has different behaviours and prognoses, requiring different treatment approaches. When diagnosed in advanced stages, it presents great resistance to therapies, with the overall survival ranging below 50–80%.

As a result, the need for precision medicine arises, aiming to adapt the whole spectrum of health care to each patient, namely in terms of the customisation or penalisation of the prevention, screening, risk stratification, therapy, and evaluation of the response to treatment. Radiomics is considered a new approach in precision medicine, which has been studied more and more in recent years. By extracting quantitative, measurable, and degradable features from medical images of interest, more information can be gained about a disease. Medical imaging is a non-invasive technique that can present a complete view of the tumour phenotype and its environment at a macroscopic level. The ambition is to link radiomics-based data with biological and clinical endpoints to enable clinical decision-making, improve diagnostic, prognostic, and predictive accuracy, and consequently, enable personalised therapy and response assessment.

Hundreds of radiomic features are generated, so a proper feature selection/extraction strategy should be adopted to reduce the dimensionality and overfitting of the predictive models. Most of these strategies are within the machine learning field, since their goal is to improve the performance of different computational models using past experiences, creating a model capable of the classification, prediction, and estimation of a situation from a selected known feature set.

In this work, different models were created in which a tumour was described by radiomic features (imaging features extracted using the *PyRadiomics* open-source package) and by clinical features. The association between tumour characteristics and cancer outcomes, namely locoregional recurrences (LRs), development of distant metastases (DMs), and overall survival (OS), was assessed, and predictive models were created.

This study allowed conducting a brief comparison between different machine learning algorithms, namely MLP and XGBoost, and analysing the influence of variables for the construction of the models, in particular the clinical variables.

The examined clinical variables were gender, age, location of the primary tumour, T-Stage, N-Stage, TNM-Stage, HPV status, surgery, and treatment, as well as information about the times, in days, from diagnosis to the start of treatment and to the last follow-up and the time, in days, of treatment. The results obtained by applying the MLP and XGBoost algorithms demonstrated that adding the aforementioned clinical variables and times to the radiomic features extracted from the medical images was an important step towards the success of the predictive models. This was corroborated by the fact that the best results for LR, DM, and OS predictions were obtained with these variables, especially when the XGBoost algorithm was applied. For LR prediction, the AUC value increased from 56 to 74 using XGBoost, with age being a determining factor. In the prediction of DMs and OS, the AUC values increased from 67 to 84 and 75 to 91, respectively. The fundamental feature for the prediction of DMs was the time, in days, from diagnosis to the last follow-up. Finally, for the construction of the decision trees of the OS prediction model, the most significant clinical variables were the presence of distant metastases, the location of the primary tumour, and the time, in days, from diagnosis to the start of treatment and to the last follow-up.

Although the created models achieved promising results and their performance was better than the benchmark studies, the sample size was not sufficiently large to reach solid conclusions. The main conclusion of this study was that the studied cancer outcomes can indeed be expressed through CT scans. Thereby, in the future, the proposed methodology should be repeated using a larger sample size. A crucial issue that should be enhanced is the tumour segmentation [37], since the more accurate it is, the more reliable the results are. On the other hand, a key point to be investigated in the future is applying the models created using deep learning techniques [38], since they may exceed the results presented in the present study. For future studies, the split should be randomised into two groups instead of splitting them by hospital group. This should lead to a further objective validation and verification process compared to the identical data origin. It would also be valuable to statistically verify the relationship between each feature and the target variable, in order to understand which features are stronger predictors and verify if they correspond to the results achieved in this study. Additionally, it would be interesting to also apply all the mentioned strategies to PET scans. Finally, even when the AUC value of the cohort is acceptable, it could perhaps be improved if the hyperparameters were determined with a nested cross-validation methodology putting together all the patients with an inner loop to select the model hyperparameters and an outer loop to assess the model performance (randomly splitting, for example, the outer loop into 10 equal folds and the inner loop into five folds).

## Figures and Tables

**Table 1:** Global results (AUC values) of Locoregional Recurrence (LR), Distant Metastasis (DM) and Overall Survival (OS) predictions for the imaging features’ models.

	Imaging Features				
	**MLP**	**XGBoost**	**Log.R**	**RF**	**DT**
Locoregional Recurrences	0.5261	0.5765	0.4911	0.4911	0.4286
Distant Metastasis	0.6201	0.8273	0.7463	0.6275	0.5013
Overall Survival	0.7379	0.8462	0.7337	0.5857	0.5759

MLP: MultiLayer Perceptron, XGBoost: eXtreme Gradient Boosting, Log.R: Logistic Regression, RF: Random Forest, DT: Decision Tree.

**Table 2:** Global results (AUC values) of LR, DM, and OS predictions for the imaging features + clinical data (Experiment 1) models.

	Imaging Features + Clinical Data (Experiment 1)				
	**MLP**	**XGBoost**	**Log.R**	**RF**	**DT**
Locoregional Recurrences	0.5472	0.7181	0.5089	0.5000	0.5268
Distant Metastasis	0.6318	0.8246	0.6991	0.5803	0.6518
Overall Survival	0.7157	0.8369	0.6811	0.5428	0.5862

**Table 3:** Global results (AUC values) of LR, DM, and OS predictions for the imaging features + clinical data (Experiment 2) models.

	Imaging Features + Clinical Data (Experiment 2)				
	**MLP**	**XGBoost**	**Log.R**	**RF**	**DT**
Locoregional Recurrences	0.5626	0.7411	0.5536	0.4911	0.5357
Distant Metastasis	0.6678	0.8367	0.7024	0.6188	0.6134
Overall Survival	0.7529	0.9051	0.7172	0.5330	0.5857

**Table 4:** Comparison of the performances of the predictive models of LRs, DMs, and OS developed in the present study and in the studies of Vallières et al. (only with information of CT scans and complete work) and Diamant et al. The results presented refer to the best AUC values obtained. The present study identified with an asterisk (*) presents a more reduced dataset.

	Present Study *	Vallières et al. [11] (Only CT Scans)	Vallières et al. [11]	Diamant et al. [10]
Locoregional Recurrences	0.74	0.62	0.69	-
Distant Metastasis	0.84	0.86	0.86	0.92
Overall Survival	0.91	0.72	0.74	-

## Data Availability

The data are publicly available on The Cancer Image Archive (TCIA) [41] website and can be downloaded using the NBIA Data Retriever [42]: https://wiki.cancerimagingarchive.net/display/Public/Head-Neck-PET-CT, accessed on 11 October 2022. The source code is available on GitHub: https://github.com/MariaGoncalves3/Radiomics_for_Head_And_Neck_Cancer, accessed on 11 October 2022.

## Supplementary Materials

The following are available online at https://www.mdpi.com/article/10.3390/diagnostics12112733/s1. Part A—list of extracted features; Part B—distribution of clinical variables among the different institutions; Part C—distribution of head and neck cancer outcome data; Part D—hyperparameters. References [39,40] are cited in the Supplementary Materials.
