# Supplementary material for: Radiomics in Head and Neck Cancer Outcome Predictions
Source: Diagnostics (Basel). 2022 Nov 8;12(11):2733. doi: 10.3390/diagnostics12112733 (PMC9689406; doi:10.3390/diagnostics12112733)
Supplement: Supplementary file 1 [file diagnostics-12-02733-s001.zip › diagnostics-1995962-supplementary.pdf]

Part A- List of Extracted Features

First-order Features:

10th Percentile

90th Percentile

Energy

Entropy

Interquartile Range

Kurtosis

Maximum

Mean Absolute Deviation

Mean

Median

Minimum

Range

Robust Mean Absolute Deviation

Root Mean Squared

Skewness

Total Energy

Uniformity

Variance

Shape-based Features:

Mesh Volume

Voxel Volume

Surface Area

Surface Area to Volume ratio

Sphericity

Maximum 2D Diameter (Slice)

Maximum 2D Diameter (Column)

Maximum 2D Diameter (Row)

Maximum 3D Diameter

Major Axis Length

Minor Axis Length

Least Axis Length

Elongation

Flatness

HIGHER-ORDER FEATURES:

GLCM:

Autocorrelation

Joint Average

Cluster Prominence

Cluster Shade

Cluster Tendency

Contrast

Correlation

Difference Average

Difference Entropy

Difference Variance

Joint Energy

Joint Entropy

Informational Measure of Correlation (IMC) 1

Informational Measure of Correlation (IMC) 2

Inverse Difference Moment (IDM)

Inverse Difference Moment Normalised

Inverse Difference (ID)

Inverse Difference Normalised (IDN)

Inverse Variance

Maximum Probability

Sum Entropy

Sum of Squares

GLRM:

Grey Level Non-Uniformity

Grey Level Non-Uniformity Normalised

Grey Level Variance

High Grey Level Run Emphasis

Long Run Emphasis

Long Run High Grey Level Emphasis

Long Run Low Grey Level Emphasis

Low Grey Level Run Emphasis

Run Entropy

Run Length Non-Uniformity

Run Length Non-Uniformity Normalised

Run Percentage

Run Variance

Short Run Emphasis

Short Run High Grey Level Emphasis

Short Run Low Grey Level Emphasis

GLSZM:

Grey Level Non-Uniformity

Grey Level Non-Uniformity Normalised

Grey Level Variance

High Grey Level Zone Emphasis

Large Area Emphasis

Large Area High Grey Level Emphasis

Large Area Low Grey Level Emphasis

Low Grey Level Zone Emphasis

Size Zone Non-Uniformity

Size Zone Non-Uniformity Normalised

Small Area Emphasis

Small Area High Grey Level Emphasis

Small Area Low Grey Level Emphasis

Zone Entropy

Zone Percentage

Zone Variance

GLSZM:

Dependence Entropy

Dependence Non-Uniformity

Dependence Non-Uniformity Normalised

Dependence Variance

Grey Level Non-Uniformity

Grey Level Variance

High Grey Level Emphasis

Large Dependence Emphasis

Large Dependence High Grey Level Emphasis

Large Dependence Low Grey Level Emphasis

Low Grey Level Emphasis

Small Dependence Emphasis

Small Dependence High Grey Level Emphasis

Small Dependence Low Grey Level Emphasis

NGTDM:

Busyness

Coarseness

Complexity

Contrast

Strength

Part B- Distribution of clinical variables among the different institutions

Table S1- Distribution of clinical variables among the different institutions

Variable	Type	CHUM	CHUS	HGJ	HMR
Gender	Female<br>Male	6<br>26	10<br>24	17<br>74	9<br>29
Age	Range<br>Mean ± STD	47-82<br>66.4± 8.8	51-81<br>63.2± 7.9	18-84<br>61.1±10.9	49-85<br>62.4± 9.1
Time from diagnosis to last follow-up	Range<br>Mean ± STD	350-2012<br>1250.1±422.1	462-2841<br>1509.3±762.7	361-3402<br>1612.3±754.1	194-2136<br>1230.3±596.5
Time of treatment	Range<br>Mean ± STD	38-54<br>45.9±2.5	21-54<br>47.4±6.3	38-209<br>46.1±17.6	42-91<br>47.8±7.5
Tumour Primary Site	Hypopharynx<br>Larynx<br>Nasopharynx<br>Oropharynx<br>Unknown	0<br>0<br>3<br>24<br>5	1<br>10<br>1<br>22<br>0	4<br>14<br>14<br>55<br>4	6<br>9<br>6<br>17<br>0
T- Stage	T1<br>T2<br>T3<br>T4<br>Tx	6<br>10<br>10<br>1<br>5	3<br>11<br>14<br>6<br>0	20<br>20<br>35<br>12<br>4	2<br>16<br>9<br>10<br>1
N-Stage	N0<br>N1<br>N2<br>N3	0<br>0<br>28<br>4	13<br>6<br>15<br>0	12<br>18<br>58<br>3	4<br>4<br>25<br>5
TNM- Stage	Stage I<br>Stage II<br>Stage III<br>Stage IV<br>Unknown	0<br>0<br>1<br>28<br>3	1<br>6<br>9<br>18<br>0	1<br>5<br>28<br>57<br>0	0<br>2<br>5<br>31<br>0
HPV- Status	Positive<br>Negative<br>Unknown	10<br>1<br>21	4<br>5<br>25	30<br>30<br>31	2<br>0<br>36

Surgery	Yes<br>No<br>Unknown	0<br>0<br>32	0<br>0<br>34	11<br>80<br>0	0<br>0<br>38
Treatment	Only Radiation<br>Chemo- Radiation	0<br>32	12<br>22	5<br>86	6<br>32
Outcome	Locoregional Recurrence<br>Distant Metastasis<br>Death	6<br>3<br>3	6<br>7<br>9	12<br>16<br>14	8<br>10<br>16

To make an accurate and precise diagnosis, the physician has to evaluate the patient according to his symptoms, based on his clinical and hereditary history and a combination of tests. The exams and tests performed may vary according to the patient's complaints, however, in general, the initial evaluation consists of a combination of an inspection test, palpation, indirect mirror examination and direct endoscopy. Under suspicion of the presence of cancer, it is necessary to perform a biopsy, that is, an invasive procedure in which a tissue sample is collected for later microscopic analysis [25],[39],[28]. Medical imaging have an important role in tumour diagnosis since they allow its visualization and evaluation.

For an initial evaluation, all patients usually undergo a Computerized Tomography (CT) or Magnetic Resonance Imaging (MRI). Patients considered to have a high metastatic risk and/or secondary malignant tumours should also undergo a Positron Emission Tomography (PET) examination. The PET examination can be used both for an initial assessment (e.g., locating primary tumours of unknown site) and for re-assessment after treatment, as it allows the identification of persistent tumours, i.e., tumour recurrences. The PET/CT combination has been highly used for tumour detection and therapeutic planning and monitoring. Technological advances in medical imaging have contributed to the oncologic advance, however, these methods still lack valuable information in the evaluation of tumour progress and involvement [25],[40].

The knowledge of the stage of the disease is paramount for the physician to plan the best treatment. Currently, oncologists use the Tumour- Node- Metastasis (TNM) classification system developed by the American Joint Committee on Cancer and the Union for International Cancer Control. The T indicates the size and extent of the primary tumour and invasive characteristics. The N evaluates the spread of regional lymph nodes. Finally, the M reveals the presence or absence of distant metastasis. In opposition to the T and M classification systems,

which are independent of the site of occurrence, the N classification system differs if the tumour has developed in the nasopharynx [25],[40],[9],[27],[29].

Patients with the same tumour location and classified with the same stage may present different responses to the same treatment and, consequently, present disparate clinical results. Thus, the choice of the best and most appropriate treatment for each case takes into consideration not only the tumour location, size, invasion, histological type and its stage, but also specific factors of the patient (e.g. his clinical status and comorbidities) [28],[27]. The decision is made jointly by oncologists and surgeons, radiation oncologists, dentists, as well as nutritionists and rehabilitation therapists and the treatment possibilities in the last 4 decades are mainly surgery, platinum and radiation-based therapies, chemotherapy, or a combination of them [28],[40],[27],[32].

Part C- Distribution of Head and Neck Cancer Outcome data

1. Locoregional Recurrences (LR)

Considering that LR=0 is representative of cases in which there are no locoregional recurrences and LR=1 is representative of cases in which there are locoregional recurrences, it is possible to verify a higher incidence of the first scenario. In training cases there are 107 and 18 cases of LR=0 and LR=1, respectively. In test cases there are 56 and 14 cases of LR=0 and LR=1, respectively.

Figure S1 shows that the data is very poorly distributed, with a tendency or class distortion.

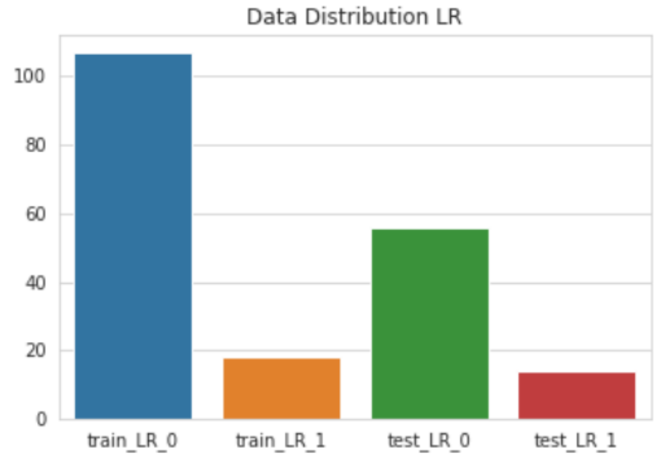

Figure S1- Data distribution for the LR prediction.

2. Distant Metastasis (DM)

Assuming that DM=0 represents cases where there are no distant metastasis and DM=1 represents cases where there are distant metastasis, in Figure S2 it is possible to verify that there is again a higher prevalence in negative cases. In training cases there are 102 and 23 cases of DM=0 and DM=1, respectively. In test cases there are 57 and 13 cases of DM=0 and DM=1, respectively.

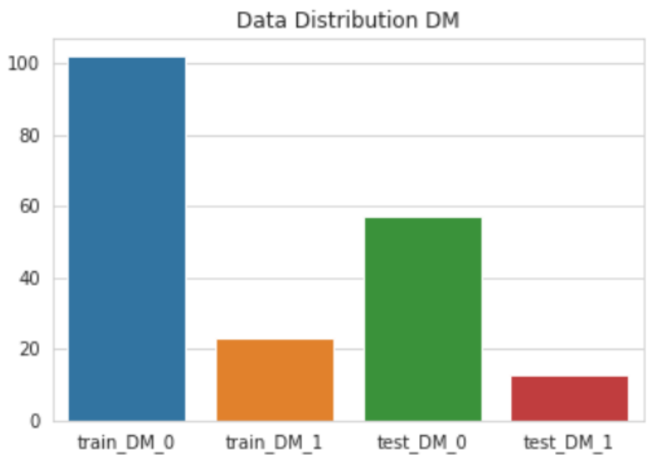

Figure S2- Data distribution for the DM prediction.

3. Overall Survival (OS)

OS=0 and OS=1 represent, respectively, the cases in which the patient survives and does not survive. In Figure S3 is, again, possible to verify a great imbalance in the data. In the training cases there are 102 and 23 of OS=0 and OS=1, respectively. In the test cases there are 51 and 19 cases of OS=0 and OS=1, respectively.

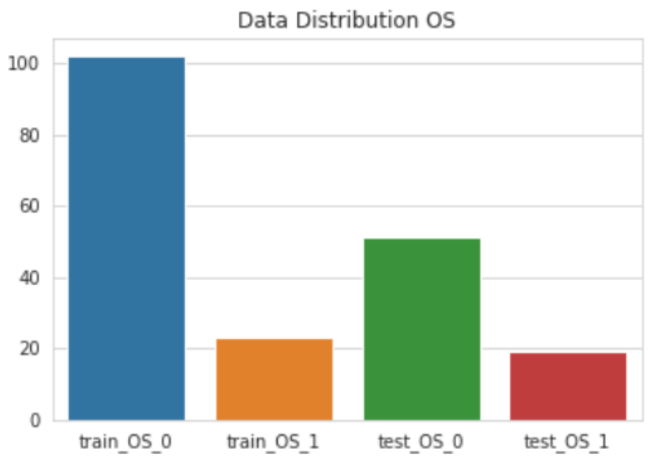

Figure S3- Data distribution for the OS prediction.

Part D- Hyperparameters

Table S2- The XGBoost hyperparameters in Experiment 2, which achieved the highest AUC values in LR, DM and Os predictions.

Hyperparameters	Locoregional Recurrences	Distant Metastasis	Overall Survival
seed	42	42	42
learning rate	0.15	0.20	0.30
N_ESTIMATORS	400	100	400
MAX_DEPTH	5	6	3
MIN_CHILD_WEIGHT	4	5	3
GAMMA	0.00	0.00	0.00
REG_ALPHA	0.40	0.00	0.20
REG_LAMBDA	0.90	0.90	0.90
SUBSAMPLE	0.80	0.40	0.90
COLSAMPLE_BYTREE	0.80	0.80	0.50
SCALE_POS_WEIGHT	16	18	18

References

[9] L. Mao, W. K. Hong, and V. A. Papadimitrakopoulou, Focus on head and neck cancer, pp. 311–316, 2004.

[25] A.C.S. Poon, K.M. Stenson. Overview of the diagnosis and staging of head and neck cancer. In: UpToDate, Post, TW (Ed), UpToDate, Waltham, MA, 2012.

[27] A.C.S. Poon, K.M. Stenson. Overview of treatment for head and neck cancer. In: UpToDate, Post, TW (Ed), UpToDate, Waltham, MA, 2015.

[28] O. Kaidar-person, Z. Gil, and S. Billan, Precision medicine in head and neck cancer, *Drug Resist. Updat.*, 2018.

[29] J. A. Brennan and D. Sidransky, Molecular staging of head and neck squamous carcinoma, pp. 3–10, 1996.

[32] J. E. Nör, J. S. Gutkind, Head and Neck Cancer in the New Era of Precision Medicine, *J. Dent. Res.*, vol. 97, no. 6, pp. 601–602, 2018.

[39] N. C. Institute, Head and Neck Cancers, 2017. [Online]. Available: https:// www.cancer.gov/types/head-and-neck/head-neck-fact-sheet. [Accessed: 10-Aug-2020].

[40] S. On, Head and Neck Cancer: Changing Epidemiology, Diagnosis, and Treatment, vol. 83, no. April, pp. 489–501, 2008.
